# Tuberculosis treatment outcomes: a fifteen-year retrospective study in Jos-North and Mangu, Plateau State, North - Central Nigeria

**DOI:** 10.1186/s12889-020-09289-x

**Published:** 2020-08-11

**Authors:** Comfort Nanbam Sariem, Patricia Odumosu, Maxwell Patrick Dapar, Jonah Musa, Luka Ibrahim, John Aguiyi

**Affiliations:** 1 Department of Clinical Pharmacy and Pharmacy Practice, Faculty of Pharmaceutical Sciences, University of Jos, Plateau State, Jos, Nigeria; 2 Department of Pharmaceutical Chemistry, Faculty of Pharmaceutical Sciences, University of Jos, Plateau State, Jos, Nigeria; 3 Department of Obstetrics and Gynaecology, Faculty of Medical Sciences, University of Jos, Plateau State, Jos, Nigeria; 4 Department of Public Health, Ministry of Health, Plateau State, Jos, Nigeria; 5 Department of Pharmacology, Faculty of Pharmaceutical Sciences, University of Jos, Plateau State, Jos, Nigeria

**Keywords:** Tuberculosis, Treatment outcomes, Retrospective study, Plateau State, Nigeria

## Abstract

**Background:**

Globally, tuberculosis (TB) is the leading cause of death from a single infectious agent. Adherence to TB therapy is an important factor in treatment outcomes, which is a critical indicator for evaluating TB treatment programs. This study assessed TB treatment outcomes using a fifteen-year record of tuberculosis patients who received treatment in Jos-North and Mangu Local Government Areas of Plateau State, North-Central Nigeria.

**Methods:**

The retrospective facility based study was done in five TB treatment centers which account for more than half of data for tuberculosis patients in Plateau State. Data were collected from 10,156 TB patient’s health records between 2001 and 2015. Treatment outcomes were categorized as successful (cured, treatment completed) or unsuccessful (non-adherent, treatment failure or death). A descriptive analysis was done to assess the factors associated with treatment outcomes. Relevant bivariate and multivariate logistic regression were done. All statistical analyses were performed on Stata version 11, College station, Texas, USA.

**Results:**

During the study period, 58.1% (5904/10156) of the TB patients who received treatment were males. The Mean age ± SD was 35.5 ± 15.5 years. The overall treatment success rate was 67.4%; non-adherence/defaulting rate was 18.5%, with majority of patients defaulting at the end of intensive phase of treatment. The sputum conversion rate was 72.8% and mortality rate was 7.5%. A decrease in successful treatment outcomes rate from 83.8% in 2001 to 64.4% in 2015 was observed. The factors associated with treatment success were gender, age, year of enrollment, and HIV status. Extrapulmonary TB was less likely associated with treatment success (AOR:95% CI- 0.72:0.61–0.84, *p <* 0.001).

**Conclusion:**

With the decrease in treatment success rates, underlying reasons for medication non-adherence and treatment failure should be resolved through adherence counseling involving the patient and treatment supporters, with education on voluntary counseling and testing for HIV among TB patients.

## Background

Tuberculosis (TB) is a bacterial infection caused by *Mycobacterium tuberculosis*. It is a major global public health concern being the tenth leading cause of death worldwide, and the leading cause of death from a single infectious agent since 2011, ahead of the Human Immunodeficiency Virus (HIV) disease [1]. Globally, 10 million people (0.13%) were estimated to have fallen ill with TB in 2017; equivalent to 132 cases per 100,000 population, with 9% of the 10 million people being HIV- positive. Death toll globally from TB disease was 1.3 million, with an additional 300,000 deaths from TB among HIV positive patients in 2017. In Nigeria, TB mortality, including HIV associated TB death was 155,000 in 2017; the second highest reported mortality globally, after India [2]. The global treatment success rate was 82% among all new TB cases [1]. TB treatment saved 53 million lives globally (including HIV positive TB patients) and 11 million lives were saved in Africa. Nigeria recorded a treatment success of 86% in 2017. However, Nigeria was 6th among the high TB burden countries after India, China, Indonesia, Philippines and Pakistan [1]. There are 30 high TB burden countries (HBCs) which collectively have about 87% of the world’s TB cases. Nigeria is among the 14 countries with overlap of high burden of TB, TB/HIV and multidrug resistant-TB (MDR-TB). HBC is defined as around 100 or more cases per 100,000 population [3]. The total TB incidence rate in Nigeria was 219/100,000 population (population of 191 million people), out of which of 14% were HIV positive [1].

In Plateau state Nigeria, the Directly Observed Therapy (DOT), which is the WHO recommended treatment strategy for TB started in 2001 with five centers and expanded to 290 centers in 2019. A summary of the treatment success rate for Plateau state from 2001 to 2010 was 72.7%, defaulting rate was 14.1% and mortality rate was 8.0% [4].

*Mycobacterium tuberculosis* is an intracellular microorganism that replicates very slowly, therefore prolonged multi-drug treatment regimen (6 months) is the recommended treatment strategy implemented through the Directly Observed Therapy (DOT) [5]. Because of this treatment regimen, medication non-adherence remains a potential challenge. Prevention of new TB infections and their progression to (TB) disease is crucial in reducing the burden of disease and death caused by TB, and in achieving the End TB Strategy targets set for 2030 and 2035, which is linked with the target of the Sustainable Development Goal-SDG; to reduce the number of TB deaths by 90% by 2030, cut new cases by 80% between 2015 and 2030, and to ensure no family is burdened with catastrophic cost due to TB [6]. Current health interventions for TB prevention include: treatment of latent TB infection (LTBI), with particular attention to children aged less than five years and HIV positive TB patients, prevention of transmission of *M. TB* through infection control especially among health workers; and vaccination of children with the Bacille Calmette-Guérin (BCG) vaccine [1].

Efforts have been made to identify factors influencing medication adherence [7–13], from which interventions [13–17] have been developed to improve adherence. This is because adherence has been shown to have profound effect on other treatment outcomes [18]. Indeed, non-adherence and mortality have been shown to account for 64 and 32% poor/unsucuccessful treatment outcomes respectively [19]. A study observed that most intervention studies targetted only adherence, but improving adherence would be more valuable if it improved clinical/treatment outcomes of the patient [20]. This study therefore focused on understanding the factors associated with treatment outcomes for TB patients, since treatment outcomes are major indicators for evaluating TB treatment programs.

In Nigeria, studies have reported trends in tuberculosis treatment outcomes for the country [1], and for different states within the country [21, 22]. However assessment of treatment outcomes in individual DOT TB facilities is lacking [23]. This will enable facility-specific interventions to be implemented. Therefore, we sought to assess the factors associated with TB treatment outcomes, with more focus on the effect HIV has on TB treatment outcomes in five DOT facilities in Jos-North and Mangu Local Government Areas of Plateau state, North-Central Nigeria through a 15- year retrospective study.

## Methods

### Study design and setting

A retrospective facility-based study of TB patient’s records from January 2001 to December 2015 was done in five DOT TB treatment facilities in Jos-North and Mangu Local Government Areas of Plateau State, North-Central Nigeria, in order to assess tuberculosis treatment outcomes. Plateau State has a land mass area of 26,899 km^2^ with a population of 3,206,531 people [24]. The TB facilities were preselected because they account for more than 50% of all TB cases in Plateau State, and had the needed health records of TB patients since the onset of the DOT programme in 2001. Demographic and patient data collected by the health workers at the facilities are usually entered into the patient’s treatment cards and the facility’s register.

The study centers were: Faith Alive Foundation Hospital (FAF), Our Lady of Apostles (OLA), COCIN Hospital and Rehabilitation Centre (CHRC) Mangu, and Bingham University Teaching Hospital (BUTH), which are faith based hospitals. Plateau State Specialist Hospital (PSSH) is a tertiary health care institution owned by Plateau State Government. CHRC, FAF and OLA are secondary health care institutions, while BUTH is a tertiary health care institution.

### Ethics

The Institutional Health Research Ethical Committee of the Jos University Teaching Hospital, Jos, Nigeria approved the protocol of this study to access the data used for the research and to use de-identified patient data for analysis.

### Data collection

Data were collected from 10,156 TB facility registers and treatment cards of patients who accessed TB treatment from DOT treatment centers from 2001 to 2015 by the researcher and trained research assistants. Data were collected manually, using a pre-designed form before tranferring to the study database. Data collected included TB patient’s demographic (sex, age, address, year of enrollment) and clinical characteristics (diagnosis, TB category, retreatment, sputum Acid Fast Bacilli-AFB analysis, period of defaulting), as well as their treatment outcomes. Socioeconomic data were not recorded at the facilities during the study period. Incomplete data especially those without recorded treatment outcomes were excluded from the study.

### Data analysis

Data checking and coding was done in Microsoft Excel before exporting to STATA® version 11.0 (College Station Texas, USA) for analysis. Demographic and clinical characteristics of the TB patients were presented in proportions. Categorical variable proportions were compared using Chi-square test and where appropriate, Fisher’s exact test was used. Bivariate analysis was used to determine patient and clinical characteristics factors associated with treatment outcomes. For the multivariate analysis, factors with *p* values ≤0.2 were included in the model, taking into account all the potential confounders. Missing values, which were determined to be missing at random were managed using complete case analysis.

### Definition of terms

Treatment outcomes of TB patients in this study were classified as successful (cured or treatment completed) or unsuccessful (defaulted, treatment failure or died), as defined from the World Health Organization (WHO) and National TB and Leprosy Control Program (NTBLCP) guidelines [1, 5, 25, 26]. This study used successful and unsuccessful treatment outcomes as the outcome measure.

**Cured** refers to a pulmonary TB patient who was smear or culture positive at the beginning of treatment and is smear or culture negative upon completion of treatment [5].

**Completed Treatment** is a TB patient who completed treatment but without evidence (no laboratory test) at the end of treatment [25].

**Successful treatment outcome:** Cured and completed treatment together make up successful treatment outcomes which should increase towards 100% and reach at least 85% with good case management [5].

**Unsuccessful Treatment outcome** includes: defaulted/lost to follow-up, treatment failure or died.

**Treatment Failure** is a PTB patient who was smear or culture positive at beginning of treatment and remains positive at month 5 or later during their most recent course of treatment [5].

**Lost to follow-up** is a TB patient who did not start treatment or whose treatment was interrupted for two consecutive months or more [5].

**Defaulted** is defined by the WHO as missing more than 20% of the prescribed doses during the treatment period i.e. a treatment interruption of two consecutive months or more after at least 1 month on treatment [26]. The definition of defaulters however can vary within national programs, for example, the Federal Ministry of Health in Nigeria, defined defaulting as not taking anti-TB medications consecutively for more than 2 days intensive phase and more than two consecutive weeks continuation phase [25].

**Retreatment** is a sputum AFB positive TB patient who had a one or more month extension of intensive phase due to sputum inconversion [25].

**Relapse** is a patient who was cured or completed treatment, but returned sputum positive or with clinical symptoms of TB (either a true relapse or a new episode of TB caused by reinfection) [5].

**Died** refers to a TB patient who died for any reason during the course of treatment [25].

## Results

During the study period we utilized data from 10,156 TB patients who received treatment from five treatment centers. The mean age ± SD was 35.5 ± 15.5. The proportion of males was more (58%) than the females as seen in Table 1. Majority of the TB patients were in the productive age of 24–35 years of age (33.4%) and were HIV positive (38.4%). Patient enrollment increased from 22.0 to 39.6% over the 15-year period, with the enrollment of HIV positive TB patients also increasing from 2.3 to 57.7%. Mean time-outcome ± SD was 5.4 ± 3.0 (time in months).

**Table 1:** Demographic Characteristics of TB Patients by HIV Status (*n* = 10,156)

Variable	HIV Negative (*n* = 3733) Freq. (%)	HIV Positive (*n* = 2968) Freq. (%)	Unknown HIV Status (*n* = 3455) Freq. (%)	Total Freq. (%)
**Sex**				
Male	2467 (66.1)	1343 (45.2)	2094 (60.6)	5904 (58.1)
Female	1266 (33.9)	1625 (54.8)	1361 (39.4)	4252 (41.9)
**Age (Years)**				
0–14	159 (4.3)	93 (3.1)	297 (8.6)	549 (5.4)
15–24	503 (13.5)	237 (8.0)	261 (7.6)	1001 (9.9)
25–34	1135 (30.4)	1069 (36.0)	599 (17.3)	2803 (27.6)
35–44	686 (18.4)	856 (28.8)	431 (12.5)	1973 (19.4)
45–54	489 (13.1)	364 (12.3)	238 (6.9)	1091 (10.7)
> 54	583 (15.6)	163 (5.5)	230 (6.7)	976 (9.6)
Not Recorded	178 (4.8)	186 (6.3)	1399 (40.5)	1763 (17.4)
**Patient Residence**				
Jos North	1393 (37.3)	1188 (40.0)	1992 (57.7)	4573 (45.0)
Jos South	390 (10.4)	446 (15.0)	594 (17.2)	1430 (14.1)
Jos East	139 (3.7)	127 (4.3)	194 (5.6)	460 (4.5)
Other LGAs in Plateau	992 (26.6)	531 (17.9)	540 (15.6)	2063 (20.3)
Other States Outside				
Plateau	133 (3.6)	138 (4.6)	134 (3.9)	405 (4.0)
Not recorded	686 (18.4)	538 (18.1)	1 (0.0)	1225 (12.1)
**Year of Enrollment**				
2001–2005	0 (0.0)	67 (2.3)	2169 (62.8)	2236 (22.0)
2006–2010	1531 (41.0)	1188 (40.0)	1179 (34.1)	3898 (38.4)
2011–2015	2202 (59.0)	1713 (57.7)	107 (3.1)	4022 (39.6)

*Freq*. Frequency, *LGAs* Local Government Areas

**Fig. 1:**
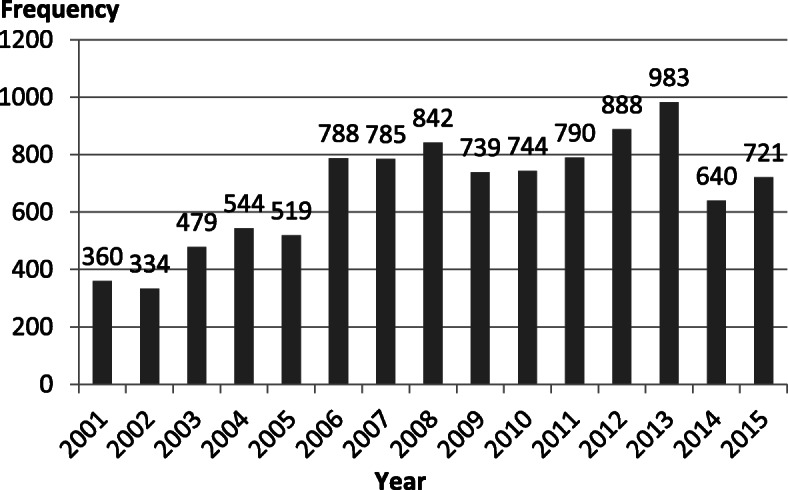
Tuberculosis Patient’s Enrollment Trend (*n* = 10,156)

Majority of the patients were new (93.3%) and had pulmonary TB (92.4%). Most of the patients that returned after default, relapsed, developed multidrug resistance, or needed retreatment were HIV negative TB Patients. Non-adherence/defaulting rate was 18.5%, with majority of the patients, especially those with unknown HIV status defaulting at the end of intensive phase. The sputum conversion rate was 72.8% (Table 2). The patient enrollment in 2001 was 360 patients, which rose to a peak of 983 patients in 2013, but decreased to 721 in 2015 as shown in Fig. 1.

**Table 2:** Clinical characteristics of tuberculosis patients by HIV Status (*n* = 10,156)

Variable	HIV Negative (*n* = 3733) Freq. (%)	HIV Positive (*n* = 2968) Freq. (%)	Unknown HIV Status (*n* = 3455) Freq. (%)	Total Freq. (%)
**TB Diagnosis**				
Pulmonary TB	3311 (88.7)	2818 (94.9)	3258 (94.3)	9387 (92.4)
Extra-PTB	422 (11.3)	150 (5.1)	197 (5.7)	769 (7.6)
TB Spine	227 (6.1)	29 (1.0)	92 (2.7)	348 (3.4)
TB Adenitis	39 (1.0)	22 (0.7)	19 (0.5)	80 (0.8)
TB Abdomen	135 (3.6)	91 (3.1)	61 (1.8)	287 (2.8)
Others^a^	21 (0.6)	8 (0.3)	25 (0.7)	54 (0.5)
**TB Category**				
New	3405 (91.2)	2778 (93.6)	3290 (95.2)	9473 (93.3)
RAD/TALF	104 (2.8)	59 (2.0)	55 (1.6)	218 (2.1)
Relapse	161 (4.3)	72 (2.4)	49 (1.4)	282 (2.8)
Trt. Failure	11 (0.3)	7 (0.2)	1 (0.0)	19 (0.2)
Transfer-In	52 (1.4)	52 (1.8)	60 (1.7)	164 (1.6)
**Needing Retreatment**				
No	3290 (88.1)	2704 (91.1)	2979 (86.2)	8973 (88.4)
Yes	282 (7.6)	136 (4.6)	104 (3.0)	522 (5.1)
Indeterminate^b^	161 (4.3)	128 (4.3)	372 (10.8)	661 (6.5)
**Sputum AFB on Diagnosis**				
Sputum Negative	2355 (63.1)	2398 (80.8)	2480 (71.8)	7233 (71.2)
Sputum AFB Positive	1378 (36.9)	570 (19.2)	975 (28.2)	2923 (28.8)
**Sputum AFB After Intensive Phase**				
Sputum Negative	3510 (94.0)	2806 (94.5)	3044 (88.1)	9360 (92.2)
Sputum AFB Positive	39 (1.0)	18 (0.6)	12 (0.3)	69 (0.7)
Indeterminate^b^	184 (4.9)	144 (4.9)	398 (11.5)	726 (7.1)
**Defaulting Period (*n*** **= 1874)**				
During Intensive Phase	97 (2.6)	65 (2.2)	271 (7.8)	433 (4.3)
End of Intensive Phase	190 (5.1)	191 (6.4)	559 (16.2)	940 (9.3)
Continuation Phase	96 (2.6)	62 (2.1)	343 (9.9)	501 (4.9)
**Binary Outcome**				
Unsuccessful	919 (24.6)	781 (26.3)	1599 (46.3)	3299 (32.5)
Successful	2814 (75.4)	2187 (73.7)	1855 (53.7)	6856 (67.5)

^a^=Disseminated TB, Miliary TB, Ovarian TB, Pleural TB, Skin TB, Heart TB

^b^Indeterminate due to lost to follow-up or death during intensive phase of treatment *RAD* Return after Default, *TALF* Treatment after lost to follow up, *Trt* Treatment

Treatment outcomes from DOT Facilities showed treatment success rate highest in OLA hospital (86.2%) Non-adherence/defaulting rate was highest in Plateau State Specialist Hospital (26.4%). Mortality rate was highest (10.5%) in CHRC Mangu (Table 3).

**Table 3:** Treatment Outcomes from DOT Facilities (*n* = 10,156)

Hosp.	Successful Treatment Freq. (%)	Defaulted Freq. (%)	Treatment Failure Freq. (%	Died Freq. (%)	Discontinued Treatment Freq. (%)	TransferOut Freq. (%)	Total
OLA	1189 (86.2)	58 (4.2)	15 (1.1)	79 (5.7)	2 (0.2)	36 (2.6)	1379 (13.6)
FAF	1126 (85.6)	81 (6.2)	4 (0.3)	84 (6.4)	0 (0.0)	20 (1.5)	1315 (12.9)
CHRC	1467 (77.3)	55 (2.9)	4 (0.2)	199 (10.5)	1 (0.1)	172 (9.1)	1898 (18.7)
PSSH	1872 (58.2)	848 (26.4)	17 (0.5)	236 (7.3)	11 (0.3)	233 (7.2)	3217 (31.7)
BUTH	1190 (50.7)	846 (36.1)	11 (0.5)	161 (6.9)	4 (0.2)	135 (5.8)	2347 (23.1)
Total	6844 (67.4)	1888 (18.6)	51 (0.5)	759 (7.5)	18 (0.2)	596 (5.9)	10,156 (100.0)

*Hosp.* Hospital, *Freq.* Frequency, *OLA* Our Lady of Apostles Hospital, *FAF* Faith Alive Foundation Hospital, *CHRC* COCIN Hospital and Rehabilitation Centre Mangu, *BUTH* Bingham University Teaching Hospital, *PSSH* Plateau State Specialist Hospital

TB patients with known HIV status had higher treatment success rates than those with unknown HIV status. Non-adherence/defaulting rate was highest (34.0%) among TB patients with unknown HIV status and mortality was highest (11.2%) among HIV positive TB patients (Table 4).

**Table 4:** Distribution of Tuberculosis Treatment Outcomes by HIV Status (*n* = 10,156)

Treatment Outcome	HIV Negative Freq. (%)	HIV Positive Freq. (%)	Unknown HIV Status Freq. (%)	Total Freq. (%)
Transfer-Out	227 (38.2)	117 (19.7)	251 (42.2)	595 (5.9)
Cured	1037 (50.8)	488 (23.9)	514 (25.2)	2039 (20.1)
Treatment Completed	1777 (36.9)	1699 (35.3)	1341 (27.8)	4817 (47.4)
Treatment Success	2814 (41.0)	2187 (31.9)	1856 (27.1)	6857 (67.5)
Defaulted	383 (20.4)	318 (17.0)	1173 (62.6)	1874 (18.5)
Treatment Failure	33 (62.3)	11 (20.8)	9 (17.0)	53 (0.5)
Died	266 (35.0)	331 (43.6)	162 (21.3)	759 (7.5)
Discontinued Trt.	10 (55.6)	4 (22.2)	4 (22.2)	18 (0.2)
Total	3733 (36.8)	2968 (29.2)	3455 (34.0)	10,156 (100.0)

*Trt* Treatment, *Freq.* Frequency

**Fig. 2:**
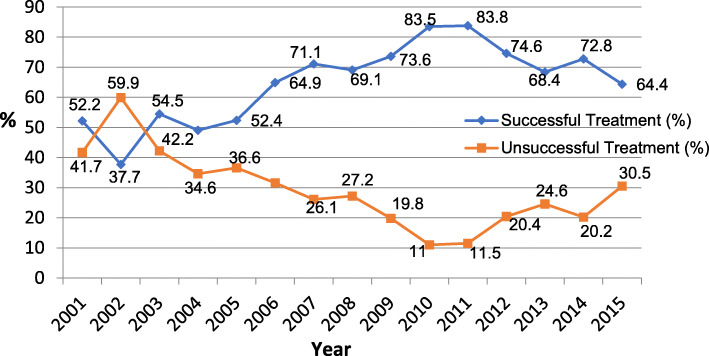
Trend of Tuberculosis Treatment Outcomes (*n* = 10,156)

The trend of TB treatment outcomes in Fig. 2 showed a steady increase in treatment success rates over the years, with a peak of 83.8% in 2011, but dropped to a low 64.4% in 2015. A consequent reverse trend was seen in unsuccessful treatment, with a peak in 2002 (59.9%) and 30.5% in 2015.

The factors significantly associated with treatment success from a univariate analysis were sex, age, year of enrollment, HIV status, TB category, retreatment and adherence. After adjusting for sex, age, year of enrollment and TB diagnosis in a multivariate analysis, having a known HIV status was more likely to be associated with treatment success than having an unknown HIV status (*p <* 0.001). Extrapulmonary TB patients were less likely to be associated with treatment success (95% CI: 0.61–0.84, *p <* 0.001) than pulmonary TB patients as shown in Table 5.

**Table 5:** Factors associated with tuberculosis treatment success

Variable	Treatment Outcome rate		COR (95% CI)	***p*** (COR)	AOR (95%CI)	***p*** (AOR)
	Unsuccessful ***n*** = 3299 Freq. (%)	Successful ***n*** = 6856 Freq. (%)				
**Gender**						
Male	1998 (60.6)	3906 (57.0)	Reference		Reference	
Female	1301 (39.4)	2950 (43.0)	1.160 (1.066–1.262)	0.010	1.161 (1.063–1.269)	0.001
**Age**						
Not Recorded	877 (26.6)	886 (12.9)	Reference		Reference	
< 15 years	243 (7.4)	353 (5.1)	1.438 (1.191–1.736)	0.001	1.289 (1.061–1.567)	0.011
> 15 years	2179 (66.1)	5617 (81.9)	2.552 (2.296–2.836)	0.001	1.819 (1.609–2.057)	0.001
**Enrollment Year**						
2001–2005	1121 (34.0)	1114 (16.2)	Reference		Reference	
2006–2010	1082 (32.8)	2816 (41.1)	2.619 (2.349–2.919)	0.001	1.646 (1.435–1.889)	0.001
2011–2015	1096 (33.2)	2926 (42.7)	2.686 (2.411–2.993)	0.001	1.255 (1.060–1.486)	0.009
**TB Diagnosis**						
PTB	3025 (91.7)	6361 (92.8)	Reference		Reference	
EPTB	274 (8.3)	495 (7.2)	0.859 (0.737–1.002)	0.053	0.717 (0.611–0.841)	< 0.001
**HIV Status**						
Negative	919 (27.9)	2814 (41.0)	2.639 (2.388–2.917)	0.001	1.848 (1.593–2.143)	< 0.001
Positive	781 (23.7)	2187 (31.9)	2.414 (2.172–2.683)	0.001	1.621 (1.394–1.885)	< 0.001
Unknown	1599 (48.5)	1855 (27.1)	Reference		Reference	
**TB Category**						
Transfer-In	40 (1.2)	124 (1.8)	Reference			
New	3063 (92.8)	6409 (93.5)	0.675 (0.471–0.966)	0.032		
RAD/TALF	86 (2.6)	132 (1.9)	0.495 (0.316–0.775)	0.002		
Relapse	99 (3.0)	183 (2.7)	0.596 (0.387–0.919)	0.019		
Trt. Failure	11 (0.3)	8 (0.1)	0.235 (0.088–0.624)	0.004		
**Needing Retreatment**						
No	2459 (74.5)	6513 (95.0)	Reference			
Yes	180 (5.5)	342 (5.0)	0.717 (0.595–0.864)	0.001		
Indeterminate+	660 (20.0)	1 (0.0)	0.001 (0.000–0.004)	0.001		
**Adherent**						
No	3296 (99.9)	15 (0.2)	0.001 (0.000 > 1.0E12)	0.999		
Yes	3 (0.1)	6842 (99.8)	Reference			

*COR* Crude Odds Ratio, *AOR* Adjusted Odds Ratio, *Trt* Treatment, *PTB* Pulmonary TB, *EPTB* Extrapulmonary TB, *RAD* Return after Default, *TALF* Treatment after lost to follow up, + Indeterminate due to lost to follow-up or death during intensive phase of treatment

## Discussion

This study was conceptualized to assess and understand the factors associated with treatment outcomes in TB patients who received treatment at selected treatment facilities in Plateau State, North-central Nigeria, with more focus on the effect HIV has on the TB treatment outcomes. An overall treatment success rate of 67.4% was found and this was less than Plateau State’s success rate of 72.7% [4] and global success rate of 82.0% [1]. A drop in the overall treatment success rate from 83.8% in 2011 to 64.4% in 2015 was also observed. Known HIV status was significantly associated with successful treatment outcomes, while extrapulmonary TB was less likely to be associated with treatment success. The association between positive HIV status and treatment success could be attributed to better medication adherence given that most HIV infected patients in the treatment facilities typically receive intensive adherence counseling for their antiretroviral medications including the need to be adherent to TB therapy.

Tuberculosis disease was found more in males than females as similarly observed in other studies [27, 28]. The possible reasons given were; women experiencing barriers to service access, longer clinical delays in diagnosis or producing sputum of poor quality than men [29]. A community based intervention study however reported significantly more women diagnosed with TB at community level than in the health facilities because the interventions reduced barriers to services with poor women who had previously faced difficulties travelling to health centres particularly benefitting [29]. Females were more likely to have successful treatment outcomes than males as seen in Table 5 probably due to higher risk behaviours (alcohol, substance and tobacco abuse) observed in males than females [4, 27].

TB was found more in the productive age group (sexually active group), particularly among HIV positive TB patients as similarly observed in other studies and consistent with global epidemiological findings [21, 30–34]. Majority of the patients were from Jos North Local Government Area of Plateau State, where most of the data was collected. These centers were chosen because they had records of TB patients from the inception of the Directly Observed Treatment progam for TB in Plateau State in 2001. They also constitute about more than half of the population of TB patients in Plateau state.

CHRC Mangu, a rural DOT secondary facility had the lowest failure rate (0.2%) and defaulting rate (2.9%) but recorded the highest transfer-out (9.1%) and mortality rates (10.5%) than the urban DOT facilities. Training of health staff and treatment supporters should be encouraged so the community is more aware and educated on tuberculosis disease in order to increase case detection and decrease late reporting when the disease is advanced. Medication education and adherence counseling would further reduce treatment failure and defaulting rates. CHRC Mangu was receiving support from the Netherlands TB and Leprosy Relief. This drew a lot of patients from both Mangu and other Local Government Areas around the state because it was an active TB diagnostic and treatment centre. The Netherlands support was however withdrawn in 2016, now making Mangu more of a dignostic and less of a treatment centre. Therefore patients diagnosed with TB in Mangu were referred to DOT centers closest to their residence for treatment; the probable reason behind the high transfer-out rates.

### Patient enrollment and treatment outcomes

The number of TB patients that accessed treatment increased from 360 patients in 2001 to 983 in 2013, especially among HIV positive TB patients. This could likely be due to the increase in prevalence (from 2.2% in 1991 to 25% in 2010) of HIV disease among TB patients [34] and improved documention processes. DOT expansion resulting to an increase in DOT facilities from 5 in 2001 to 290 in 2019 may have also increased the case detection rate [4, 30, 31]. The number of TB patients that accessed TB treatment in the DOT centres however dropped between 2014 and 2015, probably for lack of training and update on appropriate management of TB. The trend in treatment outcomes followed a similar pattern where the treatment success increased steadily from 2001 (52.2%) to a peak in 2011 (83.8%) but dropped to 64.4% in 2015. The increase in treatment failure and mortality rate observed from the 5-year interval of treatment outcome distribution in Table 6 may be due to the high non-adherence rate observed. The Nigerian National TB and Leprosy control programme is presently training and re-training TB DOT officers to improve TB health care services.

**Table 6:** Distribution of tuberculosis treatment outcomes by year of enrollment (*n* = 10,156)

Treatment Outcome	2001–2005	2006–2010	2011–2015	Total Freq. (%)
Transfer-Out	191 (32.1)	171 (28.7)	233 (39.2)	595 (5.9)
Treatment Success	1115 (16.3)	2816 (41.1)	2926 (42.7)	6857 (67.5)
Defaulted	792 (42.3)	724 (38.6)	358 (19.1)	1874 (18.5)
Treatment Failure	6 (11.3)	22 (41.5)	25 (47.2)	53 (0.5)
Died	128 (16.9)	158 (20.8)	473 (62.3)	759 (7.5)
Treatment Discontinued	4 (22.2)	7 (38.9)	7 (38.9)	18 (0.2)
Total	2236 (22.0)	3898 (38.4)	4022 (39.6)	10,156

There was an increase in non-adherence/defaulting rate from 30.6% in 2001 to 54.2% in 2002, which was the highest defaulting rate observed over the years. This may have been as a result of the strict compliance of DOT, where TB patients came everyday for 2–3 months (initial phase of treatment) with the DOT officers observing them take their medicines. This may have contributed to the high defaulting rate since patients could potentially get tired or experience some barrier to accessing the facilities for DOT. Some of these barriers include transportation cost, financial constraints and lack of social support system. Indeed, previous reports have offered recommendations to modify the DOT system of accessing TB treatment [35, 36]. This probably led to the susequent decrease in defaulting rate as contact tracing, community DOT and decrease in number of visits to the DOT center from daily to weekly is currently being practiced.

### Factors associated with treatment outcomes

Having a known HIV status was associated with treatment success as similarly observed in other studies [37, 38]. Patients on both anti-retroviral and anti-TB medicines were more likely to adhere because they received more adherence counseling than those taking only anti-TB medicines [37]. Socioeconomic characteristics, individual behaviour and health care worker were other factors reported to be associated with non-adherence across developing countries [38]. However, socioeconomic factors were not determined in this study due to unavailability of the records. TB patients with a history of defaulting/non-adherence were less likely to have successful treatment outcomes, consistent with findings from a previous study done in Plateau state [4] and other studies [30, 33]. A strengthening of adherence counseling is encouraged so that factors responsible can be identified and resolved. This is important because non-adherence and regular treatment interruptions can lead to the development of resistant TB, treatment failure, relapse, longer infections or even death [30] - a finding observed in this study from the time trend of treatment outcomes in Table 6, and similarly reported by WHO that Nigeria is among the 14 countries with overlap high burden of TB, TB/HIV and multidrug resistant-TB [1]. A study of the pattern of treatment outcomes over the years done in this study can also be carried out to identify specific areas in TB management that require intervention in order to improve outcomes and health services.

Having a known HIV status was more likely to be associated with TB treatment success. A possible explanation for this finding is that the TB patients initially received voluntary counseling and testing, then those with TB/HIV co-infection were likely to have received more/reinforced adherence counseling from trained adherence counselors prior to commencement of therapy, including adherence to TB therapy. As with HIV treatment, TB therapy also requires high (> 90%) adherence to facilitate cure [39]. Thus, they may be more aware of the consequences of non-adherence in TB/HIV co-infection. HIV negative patients had higher odds of treatment success than HIV positive patients, consistent with findings from Central Ethiopia, Abuja-Nigeria and Abakaliki-Nigeria respectively [30, 34, 40], where HIV positive TB patients were significantly associated with poor TB treatment outcomes due to malabsorption of anti-TB medicines, high pill burden and poor knowledge about the diseases.

HIV negative TB patients had lower mortality rates than HIV positive TB patients, similar to studies reported in other states in Nigeria [34, 40], and other countries [41–43]. Possible reasons given were: TB treatment failure or complication of HIV disease [34], late diagnosis of HIV in TB patients [40], unavailability/inaccessibility of anti-retrovirals-ARVs [44], immunosuppression [45], lack of treatment supporters [46], and other co-morbidities such as cardiovascular diseases and diabetes mellitus [42–45] in HIV positive TB patients. If ARVs are procured, supplied and dispensed without disruption, it may go a long way to reduce the high mortality rate in Nigeria [2]. Extra-pulmonary TB was less likely associated with treatment success than pulmonary TB patients. This was explained in previous studies that extra- pulmonary TB may be associated with immunosuppression more than pulmonary TB [47–49].

This study was limited by missing variables not recorded in the TB registers such as education, occupation and income, making it difficult to estimate the socioeconomic status of TB patients and how it may have influenced treatment outcomes in the analysis. Also, we treated treatment outcome as binary (successful versus unsuccessful) in our logistic regression models mainly because there were small numbers in the unsuccessful treatment outcome category. We acknowledge that this simplistic categorization may have biased or obscured the effect of the association on the outcomes reported. However, this study was able to identify factors associated with TB treatment success in the selected DOTs facilities, which may help the TB programme in the state and other areas with a high burden of TB during planning and implementation of interventions to improve treatment outcomes.

## Conclusions

In summary, findings from this research showed that determination of HIV status for TB patients was associated with treatment success. HIV negative patients had higher odds of treatment success than HIV positive patients. Extra-pulmonary TB was less likely associated with TB treatment success. TB treatment outcomes from five DOT facilities were evaluated to show a decrease in tuberculosis treatment success rates from a fifteen-year retrospective study. Appropriate interventions that would strengthen the treatment supporter and health system through medication education and adherence counseling, as well as increased education on voluntary counseling and testing of HIV among TB patients is advocated. Similar studies can be carried out in other DOT facilities to identify factors associated with treatment outcomes so that appropriate facility-specific intervention(s) can be designed and implemented.

## Data Availability

The datasets generated and analyzed during the current study are available from the corresonding author on reasonable request.

## Abbreviations

TB: Tuberculosis
MDR-TB: Multidrug Resistant-Tuberculosis
AFB: Acid Fast Bacilli
NTBLCP: National Tuberculosis and Leprosy Control Program
WHO: World Health Organization
PTB: Pulmonary Tuberculosis
TALF: Treatment after lost to follow up
RAD: Return after Default
DOT: Directly Observed Therapy
OLA: Our Lady of Apostles Hospital
FAF: Faith Alive Foundation Hospital
CHRC: COCIN Hospital and Rehabilitation Centre Mangu
BUTH: Bingham University Teaching Hospital
PSSH: Plateau State Specialist Hospital
